# Role of glutathione redox system on the susceptibility to deoxynivalenol of pheasant (*Phasianus colchicus*)

**DOI:** 10.1007/s43188-019-00006-4

**Published:** 2019-11-26

**Authors:** Csaba Fernye, Zsolt Ancsin, Márta Erdélyi, Miklós Mézes, Krisztián Balogh

**Affiliations:** 1 Department of Nutrition, Faculty of Agricultural Environmental Sciences, Szent István University, Páter Károly u. 1, Gödöllő, 2100 Hungary; 2 MTA-KE-SZIE Mycotoxins in the Food Chain Research Group, Kaposvár University, Guba Sándor u. 40, Kaposvár, 7600 Hungary

**Keywords:** Lipid peroxidation, Antioxidant defense, Malondialdehyde, Reduced glutathione, Glutathione peroxidase

## Abstract

There are only a few reports on the effects of mycotoxins on pheasant (*Phasianus colchicus*) and the susceptibility to deoxynivalenol of these birds have never been reported before. The present experiment focuses to investigate the effects of different dietary concentrations of deoxynivalenol on blood plasma protein content, some parameters of lipid peroxidation and glutathione redox system and on the performance of pheasant chicks. A total of 320 1-day-old female pheasants were randomly assigned to four treatment groups fed with a diet contaminated with deoxynivalenol (control, 5.11 mg/kg, 11.68 mg/kg and 16.89 mg/kg). Birds were sacrificed at early (12, 24 and 72 h) and late (1, 2 and 3 weeks) stages of the experiment to demonstrate the oxidative stress-inducing effect of deoxynivalenol. Feed refusal was dose dependent, especially in the last third of the trial, but only minor body weight gain decrease was found. Lipid-peroxidation parameters did not show dose-dependent effect, except in blood plasma during the early stage of the trial. The glutathione redox system, reduced glutathione content and glutathione peroxidase activity, was activated in the liver, but primarily in the blood plasma. Glutathione peroxidase activity has changed parallel with reduced glutathione concentration in all tissues. Comparing our results with literature data, pheasants seem to have the same or higher tolerance to deoxynivalenol than other avian species, and glutathione redox system might contribute in some extent to this tolerance, as effective antioxidant defence against oxidative stress.

## Introduction

Trichothecenes are a group of mycotoxins having over 180 structurally related compounds [1, 2] primarily produced by *Fusarium* molds. All of these toxins characterized by a tetracyclic 12, 13- epoxytrichothec-9-ene skeleton and an olefinic bond with different side chain substitutions [3]. The clinical signs of trichothecene toxicity are weight loss, lower feed conversion ratio, feed refusal, emesis, bloody diarrhea, dermatitis, coagulopathy, necrosis and hemorrhage. These latter signs occur mainly in the mitotically active tissues [4]. Trichothecenes are potent inhibitors of protein synthesis due to the interaction of 60S ribosomal subunit in the eukaryotic cell [5]. In the trichothecene family deoxynivalenol (DON, vomitoxin) is the most prevalent mycotoxin in the world [6]. Among farm animal species swine is considered to be particularly sensitive to DON while poultry species followed by ruminants have lower sensitivity, possibly due to low bioavailability in these species [7]. This fact is supported by several literature data in which adverse effects of DON can be seen at low (1 ppm) dietary concentration in swine [8], while much higher levels even at 18 ppm [9, 10] and 66 ppm [11] have not been associated with impaired productivity in gallinaceous poultry and dairy cow, respectively. In poultry, the toxicity of DON is manifested through reduced growth rate, anemia, and decreased serum triglyceride level [12] increased relative gizzard weight [13] reduced feed intake and oral lesions [14]. It can negatively effect several egg quality parameters such as egg shell weight and albumen height [10]. High load of DON have a potential to impair immunity [15] as it results in ribotoxic stress response due to inhibiting the expression genes encoding pro-inflammatory cytokines [16]. In broiler chicken, DON is confirmed to be associated with formation of oxygen free radicals, therefore it can induce membrane and DNA damage [17]. It is clear that mycotoxin research is out of the scope of the experiments focuses on game bird species. Only the toxic effects of aflatoxin B1, ochratoxin A and T-2 toxin in partridge [18], pheasants [19, 20] and quail [21] are described. There are information about the aflatoxin B1 and T-2 toxin tolerance of mallard ducks [22] and so far this is the only game bird where exposure to DON have been studied [23]. According to the results of Boston et al. [23], mallard ducks do not avoid DON contaminated cereal grain (5.8 ppm DON) and no adverse clinical or pathological effect could be confirmed due to the consumption of contaminated diet. Since the intensity of growth is a major factor associated with susceptibility to DON [9, 12, 13], hypothetically, higher tolerance can be expected in a relatively slow growing game bird. However, DON toxin tolerance of the pheasant (*Phasianus colchicus*) has never been reported before. The purpose of present study was to investigate the effects of short and long term DON exposure on the birds’ performance, rate of lipid-peroxidation and on the antioxidant defense system of young female pheasants.

## Materials and methods

### Experimental birds and diet

A total of 320 day-old female pheasants were purchased from a hatchery and transported to the experimental facility of the Department of Nutrition, Szent István University. Birds were housed in 1 m diameter rounded wall pens. Feeding was based on a commercial pheasant diet, which was supplied in mash form. During the experiment the feed and drinking water were provided ad libitum. Feed consumption was recorded daily for each group, while individual live weight was measured before the extermination of the birds. All of the birds were exterminated by cervical dislocation and exsanguination.

### Mycotoxin production and experimental contamination of diet

In the experimental period feed was artificially contaminated with deoxynivalenol. DON produced by *Fusarium graminearum* (NRRL 5883) strain on corn substrate [24]. The basal diet (crude protein: 23.88%, crude fiber: 3.90%, crude ash: 8.30%) was artificially contaminated with ground corn containing 16,324 mg/1000 g DON toxin to reach the desired toxin concentrations. Predicted toxin levels were 5, 10, and 20 mg/kg feed in the three treatment groups, and the measured DON content of the feed was determined according to Pussemier et al. [25] by HPLC method with fluorescence detection after immunoaffinity cleanup (Table 1).

**Table 1:** Predicted and measured DON toxin concentrations of the diets (mg/kg)

Groups	Predicted	Measured
Control	0	< 0.05
Low dose	5	5.11
Medium dose	10	11.68
High dose	20	16.89

### Ethical issues

The experiments were carried out according to the regulations of the Hungarian Animal Protection Act, in fulfillment with the rules of EU. The experimental protocol was authorized by the Food Chain Safety, Land use, Plant and Soil Protection and Forestry Directorate of the Pest County Governmental Office (PE/EA/1965-7/2017).

### Experimental design

A total of 320 day old female pheasant were randomly assigned to four groups with different concentration of DON toxin (control, Low dose DON, Medium dose DON and High dose DON) with two replicates (8 pens with 40 birds in each pen). The intended toxin levels in feed (5 mg/kg feed, 10 mg/kg feed and 20 mg/kg feed) were based on previous works of, Kubena et al. [9], Huff et al. [12], Prelusky et al. [26] and Xu et al. [27] as in poultry the negative effects of deoxynivalenol occurred at relatively high toxin levels (10 mg/kg or higher). From day 1 to day 9 birds were allowed to get accustomed to the environment. In this period all the animals were fed with the control feed. Treatments started with the 1 week old birds and lasted for 21 days. Both short and long term effects of DON exposure were investigated as six birds from each group were sacrificed at the early (12th, 24th and 72nd h) and the late (day 7, 14, and 21) stages of the trial. After extermination *post mortem* blood and liver samples were taken. Blood samples were centrifuged (1500 rpm) for 10 min in order to isolate plasma. Red blood cell haemolysate (1:9 v/v) was also prepared with adding ninefold volume of redistilled water to RBC. Whole liver was taken and weighed, and lower third of the large lobe was dissected and packed for further analyses. All the samples were stored at −70 °C. For further analysis, liver samples were homogenized in 1:9 v/v physiological saline.

### Lipid-peroxidation

In the fresh liver homogenate (1:9 w/v in physiological saline) conjugated dienes (CD) conjugated trienes (CT) [28], as well as malondialdehyde (MDA) concentrations were measured to analyze the state of lipid peroxidation [29]. In blood plasma and red blood cell haemolysate only the meta-stable end-product of lipid peroxidation, malondialdehyde [30] concentration was determined.

### Antioxidant system

To study the antioxidant system, some parameters of the glutathione redox system were investigated by measuring the reduced glutathione (GSH) content [31], and glutathione-peroxidase (GPx) activity [32] in the 10,000 g supernatant fraction of liver homogenate and in the blood plasma and RBC haemolysate. GSH content and GPx activity was calculated to protein content which was determined by biuret reaction [33] in blood plasma and RBC, while Folin-phenol reagent [34] was applied for the 10,000 g supernatant fraction of liver homogenates.

### Statistical analysis

Statistical analysis of the data was carried out with the use of GraphPad InStat 3.05 software (GraphPad Software, San Diego, CA, USA). First Kolmogolov–Smirnov normality test was done and based on the results either parametric one-way analysis of variance (with Tukey’s post hoc test) or non-parametric Kruskal–Wallis test (with Dunn’s post hoc test) was carried out.

## Results

### Feed intake, weight gain, and feed conversion ratio

**Fig. 1:**
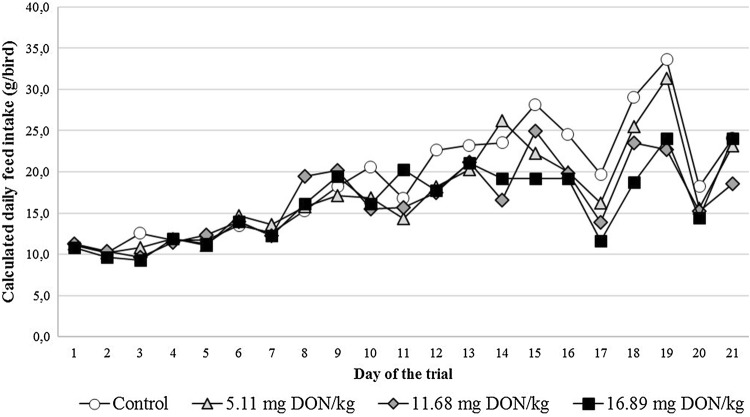
Calculated average daily feed intake (g/bird) of the pheasants in the different experimental groups throughout the feeding trial (Day 1–21)

**Fig. 2:**
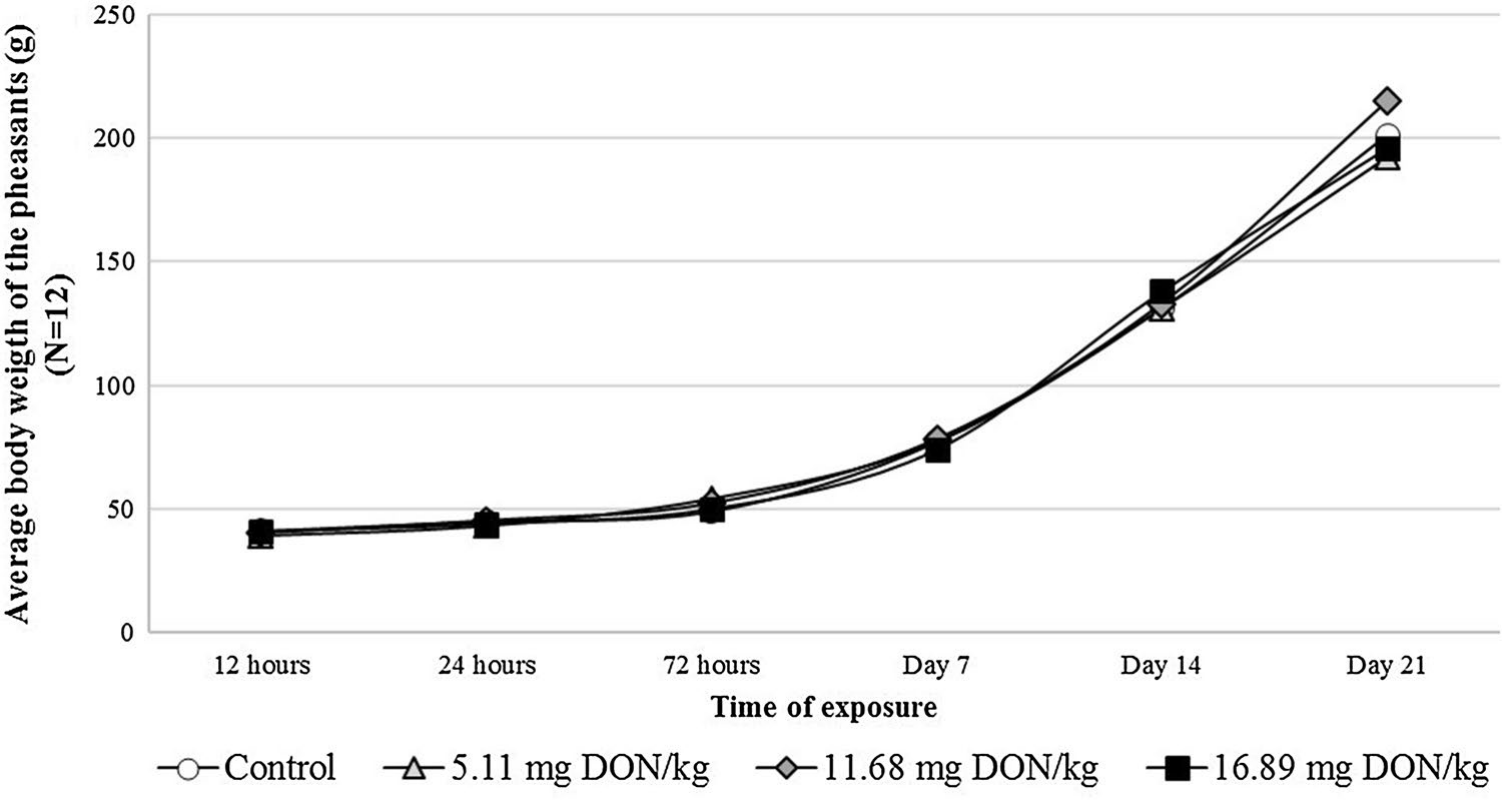
Average body weights of the pheasants (g) throughout the trial. The values represent the mean of 12 pheasants (6 birds/replicate)

Slight, clearly dose dependent feed refusal was revealed through the whole experiment (Fig. 1) and it was especially obvious in the last third of the trial. The average individual daily feed intake in the Low, Medium And High dose DON groups was 7.2, 10.6 and 12.1% lower than that of the control, respectively. The body weight of the birds however, was not in accordance with the trends in the feed intake, thus the highest values were measured primarily in the Medium DON group (Fig. 2). The final average body weight was 214.60 ± 26.94 g in this group, meanwhile it was 200.98 ± 33.68 g, 195.85 ± 14.60 g and 192.28 ± 30.83 g in the control, High and Low dose DON groups, respectively. These results affected the feed conversion ratio values as the lowest values were calculated in the Medium dose group (Table 2) among the experimental groups. However, at the first week of the trial the High dose group has shown the highest feed conversion ratio value.

**Table 2:** Effect of feeding DON contaminated diet on calculated feed conversion ratio and mortality through the experiment

Experimental group	Feed conversion ratio (feed consumed (g)/weight gained (g))			Total mortality (%)
	Week 1	Week 2	Week 3	
Control	2.26	2.48	2.50	1.25
Low dose (5.11 mg/kg)	2.19	2.32	2.39	2.50
Medium dose (11.68 mg/kg)	2.14	2.23	1.98	1.25
High dose (16.89 mg/kg)	2.43	2.16	2.20	1.25

### Clinical signs of toxicity and mortality

Increased mortality can not be attributed to DON toxicity as low level of mortality was seen in all four groups (Table 2). Also no clinical signs of toxicity have been found in the dissected animals.

### Changes of lipid peroxidation parameters

There were no changes in the marker of lipid peroxidation, malondialdehyde, levels in the early stages of the experiment in blood plasma. However, on the second week elevating tendency was seen in the Medium dose group as compared to the control, however the difference was not significant. Later, on the third week, MDA levels were significantly higher in the Medium and High dose groups as compared to the control (Table 3). MDA levels did not changed significantly in red blood cell haemolysate in the first week of the experiment, but on the second week higher MDA levels were observable in the Medium and High dose groups as compared to the Low dose and control groups, however, the difference was significant only as compared to the Low dose group. No further changes or tendencies were seen later (Table 3). Markers of the initial phase of lipid peroxidation, conjugated diene and triene levels, did not changed significantly through the experiment in liver homogenate, regardless of the toxin level or the time of the exposure (data not shown). After 12 h of exposure MDA content, as marker of termination phase of lipid peroxidation, was remarkably the highest in the High dose group, but the difference was statistically confirmed only for the Low and Medium dose groups. Similar tendencies were seen after 24 h of exposure, however no significant difference was found. On the third day of the experiment a marked decrease was observable in the Low dose group, however the only significant difference was found as compared to the Medium dose group (Table 4).

**Table 3:** Effect of DON exposure on malondialdehyde (MDA) levels in blood plasma. The values represent the mean ± SD (n = 12, 6 bird/replicate)

Time of exposure	Control	Low dose (5.11 mg/kg) DON	Medium dose (11.68 mg/kg) DON	High dose (16.89 mg/kg) DON
Blood plasma MDA (µmol/L)				
12 h	15.22 ± 7.45	15.95 ± 6.09	17.97 ± 5.25	20.14 ± 5.74
24 h	19.00 ± 6.05	16.99 ± 7.22	15.76 ± 5.73	19.55 ± 4.09
72 h	16.98 ± 2.65	16.41 ± 2.42	17.48 ± 2.54	17.54 ± 1.73
1 week	17.39 ± 4.27	17.43 ± 4.26	18.16 ± 2.84	17.78 ± 1.00
2 weeks	11.44 ± 1.98	9.85 ± 1.50	13.25 ± 3.81	10.62 ± 2.30
3 weeks	16.28 ± 3.11	18.70 ± 2.43	19.86 ± 2.46^*^	21.50 ± 2.48^*^
Red blood cell haemolysate MDA (µmol/L)				
12 h	30.78 ± 6.89	28.55 ± 6.21	32.43 ± 5.60	29.15 ± 4.86
24 h	31.73 ± 3.03	31.38 ± 2.46	28.95 ± 4.73	31.57 ± 4.99
72 h	25.61 ± 5.02	27.52 ± 3.15	29.14 ± 2.81	27.52 ± 2.08
1 week	31.55 ± 6.95	31.28 ± 3.36	35.46 ± 3.01	32.19 ± 2.55
2 weeks	23.89 ± 2.13	21.89 ± 1.77	25.71 ± 1.43^#^	24.05 ± 2.01^#^
3 weeks	36.85 ± 7.75	36.86 ± 4.99	36.18 ± 8.58	34.08 ± 5.82

*p < 0.05 compared to control; #*p* < 0.05 compared to 5.11 mg DON/kg; +*p* < 0.05 compared to 11.68 mg DON/kg

**Table 4:** Effect of DON exposure on malondialdehyde (MDA) level in liver homogenate. The values represent the mean ± SD (n = 12, 6 bird/replicate)

Time of exposure	Control	Low dose (5.11 mg/kg) DON	Medium dose (11.68 mg/kg) DON	High dose (16.89 mg/kg) DON
MDA (µmol/g wet weight)				
12 h	4.99 ± 1.33	4.71 ± 1.12	4.74 ± 1.26	6.04 ± 0.79^# +^
24 h	5.31 ± 2.66	6.11 ± 2.18	6.75 ± 1.84	7.82 ± 3.68
72 h	5.90 ± 3.00	4.17 ± 1.07	6.50 ± 1.50^#^	5.70 ± 1.72
1 week	5.86 ± 1.47	5.94 ± 1.57	6.38 ± 1.04	6.01 ± 1.76
2 weeks	6.43 ± 1.22	6.80 ± 1.84	6.23 ± 0.88	6.03 ± 0.96
3 weeks	5.30 ± 0.80	4.31 ± 1.37	5.42 ± 1.44	4.90 ± 1.28

*p < 0.05 compared to control; #*p* < 0.05 compared to 5.11 mg DON/kg; +*p* < 0.05 compared to 11.68 mg DON/kg

### Changes of a low molecular weight antioxidant, reduced glutathione, content

**Fig. 3:**
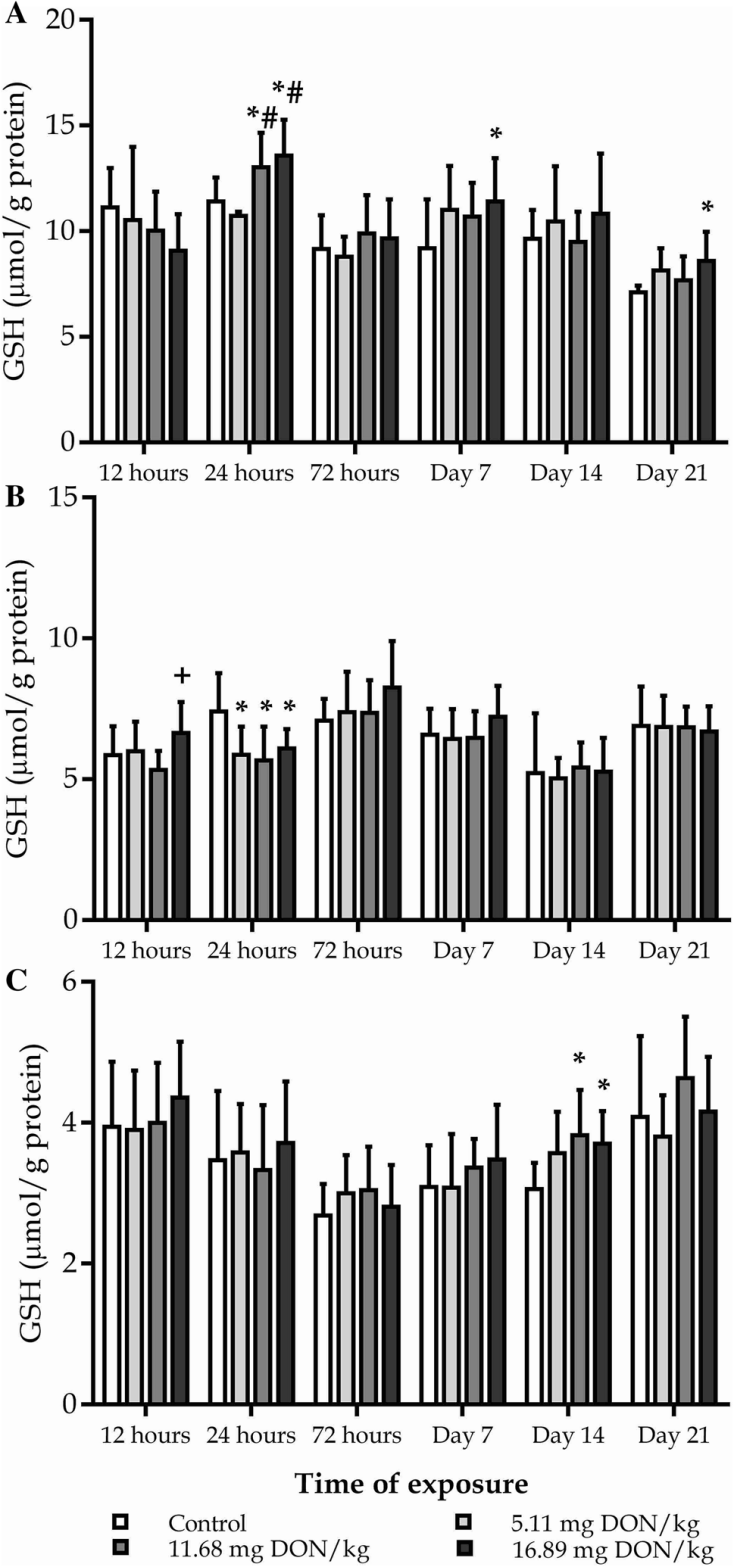
Effect of DON exposure on reduced glutathione content of blood plasma (**a**), red blood cell hameolysate (**b**) and 10,000 g supernatant fraction of liver homogenate (**c**). The values represent the mean ± SD (n=12, 6 bird/replicate). **a, b** *p<0.05 compared to control; #*p*<0.05 compared to 5.11 mg DON/kg; +*p*<0.05 compared to 11.68 mg DON/kg

The elevation of GSH levels in blood plasma in the treated groups were observable both in the early and late stages of the experiment (Fig. 3a). After 24 h of DON exposure GSH levels was significantly higher in the Medium and High dose groups as compared to the control and to the Low dose groups. Later, the GSH content increased significantly in the High dose group as compared to the control after 1 and 3 weeks of exposure (Fig. 3a). Remarkable changes in the GSH levels in red blood cell haemolysate were observable only in the early stages of the experiment (Fig. 3b). After 12 h of exposure GSH concentration was the highest in the High dose group, however, the difference was statistically significant only for the Medium dose group, and in the next 12 h GSH levels decreased significantly in all three treatment groups as compared to the control (Fig. 3b). GSH level was significantly elevated in the 10,000 g supernatant fraction of liver homogenate only after 2 week of exposure in the Medium and High dose groups as compared to the control (Fig. 3c).

### Changes of the antioxidant enzyme glutathione peroxidase

**Fig. 4:**
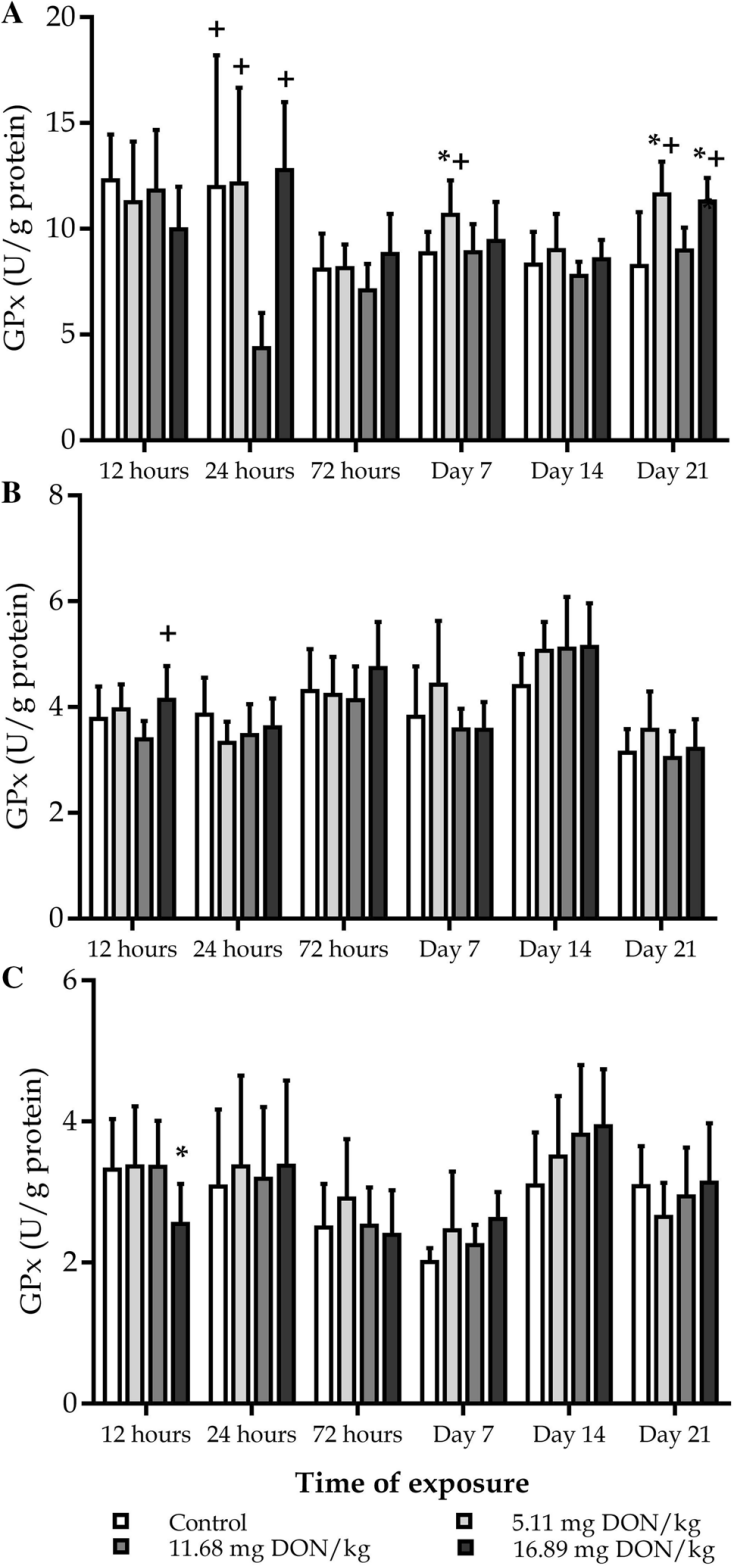
Effect of DON exposure on glutathione peroxidase activity of blood plasma (**a**), red blood cell haemolysate (**b**) and 10,000 g supernatant fraction of liver homogenate (**c**). The values represent the mean ± SD (n=12, 6 bird/replicate). **a, b** *p<0.05 compared to control; #*p*<0.05 compared to 5.11 mg DON/kg; +*p*<0.05 compared to 11.68 mg DON/kg

During the experiment the GPx activity has changed parallel with the concentration of GSH in blood plasma. Namely, GPx activity did not change significantly in the first 3 days of the experiment, but after 1 week of DON exposure elevated significantly in the Low dose group, however increasing tendency was seen in the High dose group, as compared to the control. After 3 weeks of exposure GPx activity was significantly higher in the Low and High dose groups as compared to the control (Fig. 4a). GPx activity changed similarly to GSH level also in red blood cell haemolysate, thus significantly higher GPx activity was found in the High dose group as compared to the Medium dose group, but lower enzyme activity was measured in the Low dose and the control groups (Fig. 4b). GPx activity increased significantly after 1 week of exposure in the High dose group as compared to the control in the 10,000 g supernatant fraction of liver homogenate. On the second week GPx showed increasing tendency in the two higher toxin dose groups, however no significant difference was found as compared to the control (Fig. 4c).

## Discussion

Gallinaceous poultry is known to be particularly tolerant to deoxynivalenol, therefore the consumption of high dose of DON is not necessarily accompanied with decreased weight gain [14, 35], and even significantly higher body weight was measured in chickens fed with DON contaminated diet [9]. Despite the high tolerance to deoxynivalenol there are several documented cases where high DON load affected production parameters negatively in poultry species. For instance 16 mg/kg dietary dose of DON reduced body weight gain by 8–12.4% in young broiler chickens [12, 36] and doses up to 10.24 mg/kg caused impaired growth in turkey poults [27]. Xu et al. [27] used similar doses (6.30, 10.68 and 16.97 mg/kg) of DON as in the present study and found decreased feed consumption (5.6–10.6%) and reduced body weight gain (7.7–10.1%) of broilers. According to the literature data, feed conversion ratio is generally not [9, 37, 38] or negatively affected [12, 13] by consumption of DON contaminated diet. Considering our results, moderate DON exposure has caused some reduction in the feed intake without any negative effect on weight gain. Our results on mortality are comparable with studies on broiler chicken [36, 39] as it was not influenced by the DON concentration within the range we used or the time length of exposure. Our previous study with another trichothecene mycotoxin, T-2 toxin, on young pheasant chicks with similar doses (4.22, 7.17 and 14.92 mg/kg) revealed that feed refusal in low levels were quite comparable with 5.11 mg/kg DON and 4.22 mg/kg T-2 toxin. At higher doses, however, T-2 toxin resulted considerably lower feed intake than DON. T-2 toxin also affected weight gain, feed conversion ratio and mortality more severely than DON, especially in the medium and high toxin groups [20].

Due to the DON exposure, some changes in the biochemical parameters were seen in all observed tissues, and their tendencies were similar. However, blood plasma was the most informative as most of the significant differences were found here. Elevation in the MDA concentration, occurred primarily in the two higher dose groups (11.68 and 16.89 mg/kg feed) and in the late stages of the experiment indicating that high dose and prolonged exposure are required to induce oxidative stress in pheasant. Trichothecenes known to inhibit protein synthesis [40] and the decreased level of plasma protein in the Low and Medium dose groups after 21 days of exposure is a possible sign of that effect. Similar results was found in broiler chicken after long term (42 days) exposure even with a relatively low level (3 mg/kg) of DON contamination [41].

GSH as a small molecular weight antioxidant was present in elevated concentration in blood plasma at the early and late stages of the experiment as well, and resulted parallel in increased GPx activity. Similar changes were revealed in the liver, but only at the end of the experimental period. These elevations in GSH content and GPx activity are possibly indicating that the consumed amount of DON was high enough to trigger and activate the antioxidant defense of the birds, however it was too low to maintain the activation through the whole period of the experiment. The increased GSH levels might be also the result of glutathione reductase activation due to oxidative stress induced by DON as it was revealed in another study [42], but it was not studied in our experiment. On biochemical level the major difference between T-2 toxin [20] and DON was the time of exposure required to trigger significant changes. T-2 toxin decreased protein concentration but increased GSH concentration and GPx activity from the beginning of the experiment meanwhile these effects primarily expressed after a long term (at least 1 week) of DON exposure.

It can be concluded that pheasant can tolerate short-term DON exposure even at high toxin level since most of its adverse effects such as reduced feed consumption, lowered blood protein concentration due to potential inhibition in protein synthesis or increased lipid-peroxidation have occurred only in the late stages of the experiment. It is also clear that these effects cannot be considered to be detrimental even at long term.

## Abbreviations

CD: Conjugated dienes
CT: Conjugated trienes
DON: Deoxynivalenol
GPx: Glutathione peroxidase
GSH: Reduced glutathione
MDA: Malondialdehyde
RBC: Red blood cell

## References

[1] Grove JF (1988). Non-macrocyclic trichothecenes. Nat Prod Rep.

[2] Grove JF (2000). Non-macrocyclic trichothecenes (Part 2). Prog Chem Org Nat Prod.

[3] IPCS (1990) Environmental health criteria 105. Selected mycotoxins WHO, Vammala. II: Trichothecenes. pp 71–164

[4] CAST (2003) Mycotoxins: risks in plant, animal and human systems. Task force report no. 139. Council for Agriculture, Science and Technology, Ames, IA, pp 64–65

[5] Carter CJ, Cannon M (1977). Structural requirements for the inhibitory action of 12, 13-epoxytrichothecenes on protein synthesis in eukaryotes. Biochem J.

[6] Bennett JW, Klich M (2003). Mycotoxins. Clin Microbiol Rev.

[7] Payros D, Alassane-Kpembi I, Pierron A, Loiseau N, Pinton P, Oswald IP (2016). Toxicology of deoxynivalenol and its acetylated and modified forms. Arch Toxicol.

[8] Friend DW, Trenholm HL, Elliot JI, Thompson BK, Hartin KE (1982). Effects of feeding vomitoxin-contaminated wheat to pigs. Can J Anim Sci.

[9] Kubena LF, Swanson SP, Harvey RB, Fletcher OJ, Rowe LD, Phillips TD (1985). Effects of deoxynivalenol (vomitoxin)-contaminated wheat to growing chicks. Poult Sci.

[10] Kubena LF, Harvey RB, Corrier DE, Phillips TD, Huff WE (1987). Effects of feeding deoxynivalenol (DON, vomitoxin)- contaminated wheat to female white leghorn chickens from day old through egg production. Poult Sci.

[11] Cote LM, Dahlem AM, Yoshizawa T, Swanson SP, Buck WB (1986). Excretion of deoxynivalenol and its metabolite in milk, urine and feces of lactating dairy cow. J Dairy Sci.

[12] Huff WE, Kubena LF, Harvey RB, Hagler WM, Swanson SP, Phillips TD, Creger CR (1986). Individual and combined effects of aflatoxin and deoxynivalenol (DON, vomitoxin) in broiler chickens. Poult Sci.

[13] Kubena LF, Harvey RB (1988). Response of growing leghorn chicks to deoxynivalenol-contaminated wheat. Poult Sci.

[14] Moran ET, Hunter B, Ferket P, Young LG, McGirr LG (1982). High tolerance of broilers to vomitoxin from corn infected with *Fusarium graminearum*. Poult Sci.

[15] Harvey RB, Kubena LF, Huff WE, Elissalde MH, Phillips TD (1991). Hematologic and immunologic toxicity of deoxynivalenol (DON)-contaminated diets to growing chickens. Bull Environ Contam Toxicol.

[16] Pestka JJ (2007). Deoxynivalenol: toxicity mechanisms and animal health risk. Anim Feed Sci Technol.

[17] Dvorska JE, Pappas AC, Karadas F, Speake BK, Surai PF (2007). Protective effect of modified glucomannans and organic selenium against antioxidant depletion in the chicken liver due to T-2 toxin-contaminated feed consumption. Comp Biochem Physiol C Toxicol Pharmacol.

[18] Ruff MD, Huff WE, Wilkins GC (1990). Characterization of the toxicity of the mycotoxins, aflatoxin, ochratoxin, and T-2 toxin in game birds. I: chukar partridge. Avian Dis.

[19] Huff WE, Ruff MD, Chute MB (1992). Characterization of the toxicity of the mycotoxins, aflatoxin, ochratoxin, and T-2 toxin in game birds. II: ringneck pheasant. Avian Dis.

[20] Fernye C, Ancsin Z, Bócsai A, Balogh K, Mézes M, Erdélyi M (2018). Role of glutathione redox system on the T-2 toxin tolerance of pheasant (*Phasianus colchicus*). Toxicol Res.

[21] Ruff MD, Huff WE, Wilkins GC (1992). Characterization of the toxicity of the mycotoxins, aflatoxin, ochratoxin, and T-2 toxin in game birds. III: bobwhite and Japanese quail. Avian Dis.

[22] Neiger RD, Johnson TJ, Hurley DJ, Higgins KF, Rottinghaus GE, Stahr H (1994). The short-term effect of low concentrations of dietary aflatoxin and T-2 toxin on Mallard ducklings. Avian Dis.

[23] Boston S, Wobeser G, Gillespie M (1996). Consumption of deoxynivalenol-contaminated wheat by mallard ducks under experimental conditions. J Wildl Dis.

[24] Fodor J, Németh M, Kametler L, Pósa R, Kovács M, Horn P (2006). Novel methods of Fusarium toxins’ production for toxicological experiments. Acta Agrar Kvar.

[25] Pussemier L, Piérard JY, Anselme M, Tangni EK, Motte JC, Larondelle Y (2006). Development and application of analytical methods for the determination of mycotoxins in organic and conventional wheat. Food Addit Contam.

[26] Prelusky DB, Trenholm HL, Hamilton RMG, Miller JD (1987). Transmission of (^14^C) deoxynivalenol to eggs following oral administration to laying hens. J Agric Food Chem.

[27] Xu L, Eicher SD, Apllegate TJ (2011). Effects of increasing dietary concentration of corn naturally contaminated with deoxynivalenol on broiler and turkey poult performance and response to lipopolysaccharide. Poult Sci.

[28] AOAC (1984) Official methods of analysis 28054 B, 14th edn. Arlington, USA

[29] Placer ZA, Cushman LL, Johnson BC (1966). Estimation of product of lipid peroxidation (malonyldialdehyde) in biochemical systems. Anal Biochem.

[30] Mihara M, Uchiyama M, Fukuzawa K (1980). Thiobarbituric acid value of fresh homogenate of rat as parameter of lipid peroxidation in ageing, CCl4 intoxication and vitamin E deficiency. Biochem Med.

[31] Sedlak I, Lindsay RH (1968). Estimation of total, protein-bound and non-protein sulfhydryl groups in tissues with Ellmann’s reagent. Anal Biochem.

[32] Lawrence R, Burk R (1978). Species, tissue and subcellular distribution of non Se-dependent glutathione peroxidase activity. J Nutr.

[33] Weichselbaum TE (1948). An accurate and rapid method for the determination of protein in small amounts of serum and plasma. Am J Clin Pathol.

[34] Lowry OH, Rosenbrough NJ, Farr AL, Randall RJ (1951). Protein measurement with the Folin phenol reagent. J Biol Chem.

[35] Harvey RB, Kubena LF, Rottinghaus GE, Turk JR, Caper HH, Buckley SA (1997). Moniliformin from *Fusarium fujikuroi* culture material and deoxynivalenol from naturally contaminated wheat incorporated into diets of broiler chicks. Avian Dis.

[36] Kubena LF, Huff WE, Harvey RB, Corrier DE, Phillips TD, Creger CR (1988). Influence of ochratoxin A and deoxynivalenol on growing broiler chicks. Poult Sci.

[37] Keshavarz K (1993). Corn contaminated with deoxynivalenol: effects on performance of poultry. J Appl Poult Res.

[38] Hulan HW, Proudfoot FG (1982). Effects of feeding vomitoxin contaminated wheat on the performance of broiler chickens. Poult Sci.

[39] Dȃnicke S, Matthes S, Halle I, Ueberschär KH, Döll S, Valenta H (2003). Effects of graded levels of *Fusarium* toxin-contaminated wheat and of a detoxifying agent in broiler diets on performance, nutrient digestibility and blood chemical parameters. Br Poult Sci.

[40] Kiessling KH (1986). Biochemical mechanism of action of mycotoxins. Pure Appl Chem.

[41] Faixová Z, Faix S, Bořutová R, Leng L (2007). Efficacy of dietary selenium to counteract toxicity of deoxynivalenol in growing chicken. Acta Vet Brno.

[42] Smith TK (1992). Recent advances in the understanding of Fusarium trichothecene mycotoxicoses. J Anim Sci.

